# The Scientific Contributions of Bernard Cohen (1929–2019)

**DOI:** 10.3389/fneur.2020.624243

**Published:** 2021-01-12

**Authors:** Jun Maruta

**Affiliations:** ^1^Department of Neurology, Icahn School of Medicine at Mount Sinai, New York, NY, United States; ^2^Department of Rehabilitation and Human Performance, Icahn School of Medicine at Mount Sinai, New York, NY, United States

**Keywords:** cerebellum, eye movement, spatial orientation, velocity storage, vestibuloocular reflex

## Abstract

Throughout Bernard Cohen's active career at Mount Sinai that lasted over a half century, he was involved in research on vestibular control of the oculomotor, body postural, and autonomic systems in animals and humans, contributing to our understanding of such maladies as motion sickness, mal de débarquement syndrome, and orthostatic syncope. This review is an attempt to trace and connect Cohen's varied research interests and his approaches to them. His influence was vast. His scientific contributions will continue to drive research directions for many years to come.

## Introduction

**Figure 1:**
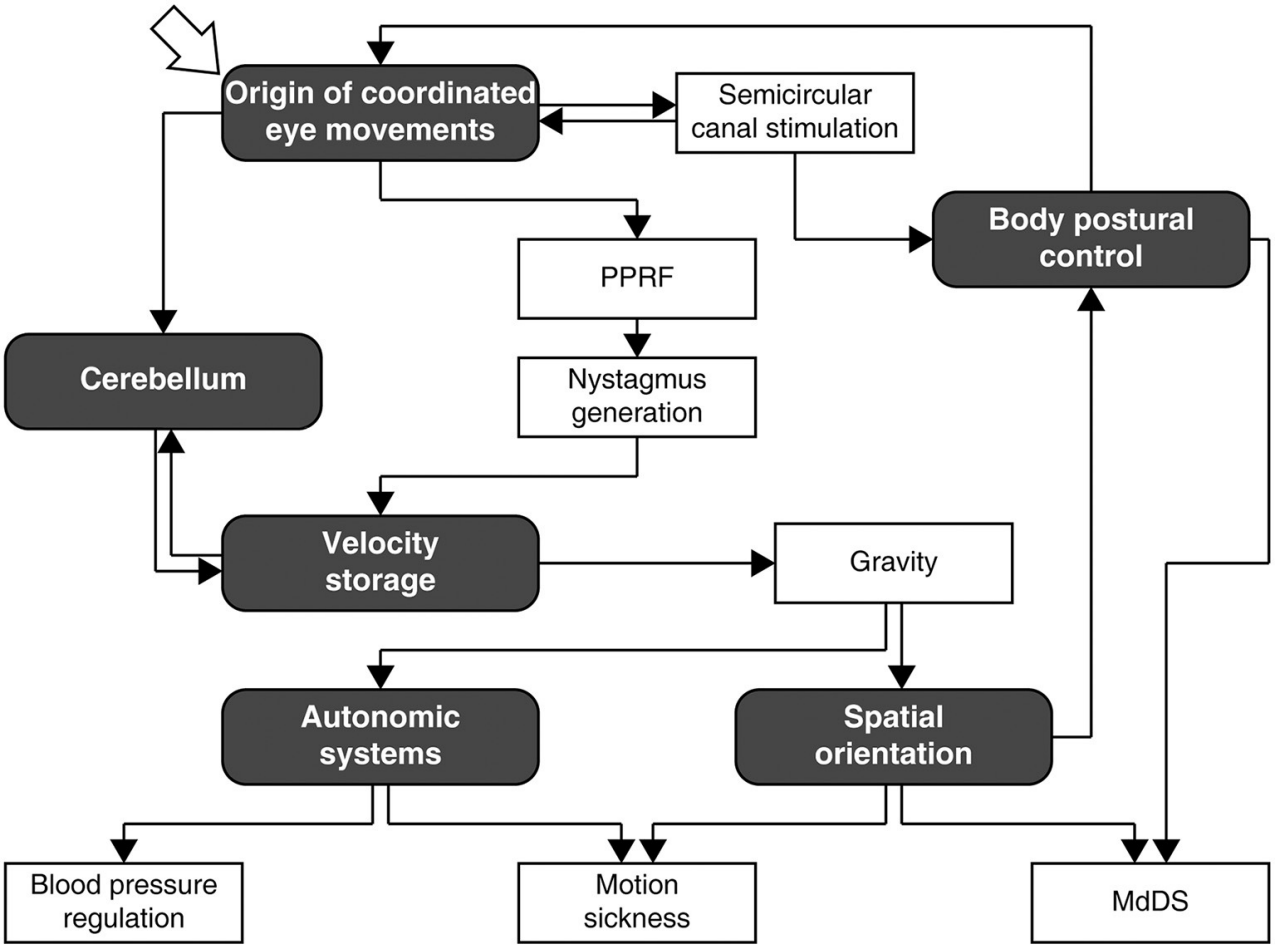
A rough navigation guide for Bernard Cohen's research pursuit as interpreted by the author. Many branches and links are omitted for simplicity of exposition. Note that the diagram does not depict neural processes involved in the mechanisms that Cohen studied, which are reviewed in depth and comprehensively elsewhere (1, 2). PPRF, paramedian pontine reticular formation; MdDS, mal de débarquement syndrome.

After education and training through Middlebury College, New York University School of Medicine, internship, residencies in neurology, and psychiatry, 2 years in the U.S. Army, and a 2-year experimental fellowship at Columbia University under Dominick Purpura, Bernard Cohen arrived at Mount Sinai Hospital in 1962 for good. Throughout his active career at Mount Sinai, he was involved in research on vestibular control of the oculomotor, body postural, and autonomic systems in animals and humans (Figure 1), and was continuously supported by grants from the NIH, NASA, the NSF, and New York City as a principal investigator. Most notably, the NIH grant entitled “The Oculomotor System and Body Postural Mechanisms” (NB00294, NS00294, EY11812), initially spearheaded by Morris B. Bender before the arrival of Cohen at Mount Sinai, ran for 50 years through 2009, an NIH record. Despite losing his central vision in both eyes circa 2012, Cohen pursued research in a full-time capacity until his retirement at the age of 88 years in 2017, thereafter he continued to be engaged in writing, mentoring, and organizing conferences. Cohen fell ill just days before the international conference born out of the Frontiers in Neurology Research Topic, “Vestibular Contributions to Health and Disease” (3), for which he was the main driving force of organization. Following a month long hospitalization, he passed away peacefully on November 27, 2019, at the age of 90 years.

## Foundation

Although only 2 years senior to Cohen, Purpura was already an established neurophysiologist by the time they crossed paths in 1960, running his own laboratory at Columbia University and having published some 40 papers by then. For 2 years with Purpura, Cohen recorded activities of thalamic and extrathalamic pathways in cats and honed his electrophysiological skills (4–6).

## Oculomotor Control

At Mount Sinai, Cohen began by identifying the eye, head, and body movements activated by the semicircular canals with Jun-Ichi Suzuki (7–9). Bender wanted Suzuki, who had just arrived from Tokyo with Fulbright support, to electrically stimulate the labyrinths. With Suzuki and Cohen respectively taking lead in surgery and electrophysiology, and aided by then-newly-available electronic technologies, the experiments were completed in 2 years (10). They were the first to demonstrate couplings of electrical stimulation of individual vestibular nerve branches to specific motor consequences. Extending from earlier pioneering studies by Lorente de Nó (11) and Szentágothai (12), Cohen and Suzuki's experiments revealed the machinery by which movement is controlled by the sum of three orthogonal vectors represented in the geometry of the semicircular canals, and their results opened a whole field of new research ranging from basic experimental, theoretical, to applied clinical (13–17).

This effort was followed by identification of the paramedian pontine reticular formation (PPRF) as the horizontal saccade premotor center, particularly in collaboration with Atushi Komatsuzaki and Volker Henn. Over the prior three decades, Bender and his colleagues had meticulously tabulated the results of stimulation and lesioning of essentially every cubic millimeter in a total volume of some 2,500 mm^3^ in the brainstem of macaque monkeys to identify the origins of bilaterally coordinated activation of ocular motor nuclei (18). Following this path, Cohen and Komatsuzaki, with Bender, succeeded in more precisely associating PPRF lesions with distinctive conjugate horizontal gaze palsies (19). Cohen continued to accumulate evidence that PPRF is responsible for production of the premotor commands for ipsilateral horizontal saccades, be they for conjugate gaze shifts or fast phases of nystagmus (20–22). Cohen and Henn then went on to uncover many of the building blocks of horizontal saccade generation with single unit recording in PPRF (23, 24). These findings vastly expanded the understanding of the structure of ocular premotor commands by isolating a distinct eye movement element and further elucidating its underlying mechanisms, much akin to a discovery and dissection of a chemical element.

Cohen and Komatsuzaki also showed that conjugate eye movements produced by stimulation of PPRF occurred at a constant velocity, with the amplitude and speed linearly dependent on the frequency of stimulation pulses delivered up to that moment (22). This finding significantly advanced not only oculomotor research but also the field of biomechanics by providing evidence for neural implementation of a mathematical integration predicted from control system theories (25).

## Velocity Storage

Cohen's next major contribution was with Theodore Raphan in quantitatively characterizing optokinetic nystagmus (OKN) and optokinetic after-nystagmus (OKAN) and formulating a mathematical model that emulated their characteristics featuring a leaky integrator (26). As noted by ter Braak as far back as in 1936 (27), OKAN, i.e., the persistence of nystagmus in darkness following a visual motion stimulus, indicates storage of motion-related signals and sustained output from this storage. Collewijn recognized the usefulness of such a storage mechanism in characterizing the slow build-up of OKN in the rabbit and conceptualized it also as a leaky integrator (28), but Cohen and Raphan put the integrator forth as the focus of the visual-vestibular interaction (29, 30), as also anticipated by ter Braak (27). Cohen's interest in this area was a natural extension of his earlier effort on saccade generation because understanding how nystagmus is shaped required ideation of a shared central processor of visual and vestibular inputs (2, 27). It had been generally assumed that there existed a brainstem mechanism that extended the time over which the vestibulo-ocular reflex (VOR) could compensate for constant-velocity head movement after the fall of the eighth nerve activity, but the conceived mechanism, shared between vestibular and visual functions, would provide a ground that could efficiently attenuate the after-response to prolonged rotation even when the vision was blocked at the stop of rotation (27, 29, 30). Cohen and Raphan termed this mechanism the velocity storage integrator.

A major impetus for this formulation was derived from fresh evidence by Waespe and Henn of vestibular nuclear activity that correlated with the strengths of nystagmus of the VOR, OKN, and OKAN in alert monkeys (31, 32) as well as from earlier results of labyrinthectomy in monkeys and rabbits, which degraded OKN and abolished OKAN (33, 34). Not surprisingly, David Robinson of the Johns Hopkins University also took note of these observations and simultaneously proposed an alternate model, which behaved equivalently to the Cohen-Raphan model but utilized a positive feedback loop in the implementation (35). While Robinson proposed his model as a demonstration of the utility of a model-based approach in understanding visual-vestibular interaction, Cohen, Raphan, and Matsuo elucidated nuances of velocity storage by testing their model against a well-defined experimental dataset (26, 29, 30). Both Cohen and Raphan, and Robinson postulated that velocity storage contributes to the sensation of self-motion (vection) (36).

Cohen and Raphan continued to characterize velocity storage as a mechanism that would facilitate ocular and postural compensation to turning motion—registration of not only the start and end of rotation but also whether one is continuing to rotate. They observed activation of velocity storage as, for example, prolonged nystagmus during the VOR to a constant velocity rotation in darkness, OKAN, and continuous unidirectional nystagmus during rotation about a tilted axis (off-vertical axis rotation, OVAR) or pitching/rolling about an earth-horizontal axis while rotating about an earth-vertical axis (pitching/rolling while rotating, PWR/RWR). Of note, the characterization of velocity storage expression to date still nearly exclusively relies on changes over time in the direction and magnitude of eye rotational velocity during slow phases of nystagmus. Two possible exceptions are subjective reporting of vection in human experiments (37, 38) and activity levels in specific classes of neurons in the vestibular nucleus in animal experiments (31, 39, 40).

Signature characteristics of velocity storage include: 1) stored rotational velocity can be discharged (“dumped”) by visual fixation or a tilt, an indication that one is no longer turning (26, 29, 30, 41, 42); 2) the ability to store velocity is permanently abolished by labyrinthectomy or vestibular nerve section (33, 43), or by cutting the commissural fibers between the bilateral vestibular nuclei (44); 3) in contrast to 2), inactivation of the semicircular canal function by plugging the duct and interrupting the flow of the endolymph, thus keeping the vestibular nerve intact, spares the velocity storage function (43); 5) the storage mechanism can be reversibly and dose-dependently inactivated by the GABA_B_ receptor agonist baclofen (45); 5) repeated vestibular stimulation can weaken, i.e., habituate, velocity storage without affecting the gain of VOR compensation during step velocity rotation, and once habituated, the state is retained for months or possibly longer (46, 47); and 6) the gain of VOR compensation can be changed up or down apparently without affecting the velocity storage capacity and is more malleable than habituation (47).

Note that tilt dumping of velocity storage occurs only in the absence of head rotation—more specifically, in the absence of head rotation about the axis with which the stored velocity is associated. In contrast, tilting the head while in rotation changes the alignment of the semicircular canals with the plane of rotation, which instead *activates* the velocity storage mechanism and, in combination with otolith input, permits reconstruction of signals related to rotation as demonstrated with PWR/RWR (41, 48–50). In further contrast, tilting the axis of rotation relative to gravity, that is OVAR (51, 52), also activates velocity storage, not through the semicircular canals but through continuous reorientation of the otoliths relative to gravity (43, 53). Incidentally, tilting the head while rotating is historically referred to as a Coriolis/cross-coupling stimulus (54), but Coriolis acceleration is not considered a significant contributor to velocity storage activation (48). On the other hand, sinusoidal translation in the plane of rotation, due to the combination of Coriolis and centripetal accelerations, tilts the gravito-inertial acceleration relative to the head without activating the semicircular canals, such that the stimulus generates a sweeping movement of linear acceleration around the head as in OVAR and similarly activates velocity storage (55).

Cohen and Raphan over time realized that velocity storage, when the head is tilted, undergoes dynamic transformation so that the axis of the output eye movement tends toward the spatial vertical as referenced by gravity (56–59). That is, the velocity storage integrator, a mechanism thought to facilitate ocular and postural compensation to rotation by representing the state of self-motion, is also equipped with orienting properties to act as a “neural gyroscope” (57, 60). This discovery led to an understanding of the three-dimensional functional configuration of velocity storage (61, 62) as well as how this configuration can contribute to the sense of relationship between self and the environment, balance, and the production of motion sickness (63, 64). The postulate that three-dimensional properties of velocity storage might be coded by vestibular-only neurons in the medial and superior vestibular nuclei was later substantiated (40).

## Spatial Orientation and Body Postural Control

The study of velocity storage led Cohen to deeply contemplate the role of the vestibular system in spatial orientation (60, 65). The term spatial orientation describes the ability to relate the position and movement of the body and body parts to spatial cues, including sensing, establishing, and maintaining connections to space (66). Simply put, spatial orientation indicates readiness for spatial interactions. Results from other laboratories indicated that, in addition to visual or vestibular input, velocity storage could be activated by somatosensory input, such as with an extended arm passively following a rotating cylindrical wall in darkness (67) or walking on a circular treadmill (68). It thus became clear that velocity storage was a focal point of not just visual-vestibular but wider multimodal sensory interaction and played an essential role in spatial orientation by providing a working memory-like function for self-motion. Earlier, Cohen with Suzuki had established the specific nature of the reflex head and postural movements produced by semicircular canal stimulation across species as a compensatory mechanism for sudden angular displacements (9), but as Cohen began to recognize the orientation property of velocity storage, he again became interested in the vestibular control of body posture and locomotion.

The seemingly mundane act of holding a stable gaze during locomotion in fact involves precise coordination of movements of the torso, limbs, head, and eyes. To achieve this feat, information about movements of these various body parts must be coded in the brain using appropriate coordinate frames. It was from this perspective that Cohen studied various strategies used by animals and humans to generate compensatory head and eye movements during circular and linear locomotion, including both angular and linear vestibulocollic reflexes and velocity storage (69–71). Furthermore, from the studies of the VOR, it had become evident to Cohen that both compensatory and orienting eye movements could be generated by the reflex arc in relation to either angular or linear acceleration (1, 65, 72, 73). As put forward by Cohen and Raphan, while compensatory eye movements support maintenance of a fixed gaze direction or gaze point in space, orienting eye movements tend to align the eye vertical with the spatial reference vector of the gravito-inertial acceleration, with a stipulation that the outcome of the VOR depends on the temporal frequency of the head movement. It was then found that corresponding compensatory and orienting head and body movements were also generated to stabilize gaze during walking, with slower orienting mechanisms controlling the dynamics of compensatory movements (74). Thus, compensatory and orienting responses should be recognized as part of generalized vestibular functions.

This line of research was also greatly relevant to space exploration, which may take place in the environment where gravity, a fundamental parameter that defines one's relationship to the environment, is not detectable. In this context, Cohen et al. focused on characterizing potentially maladaptive changes in spatial orientation and perception due to exposure to microgravity as well as whether artificial gravity produced with centrifugation could provide an effective countermeasure against these changes (75–80).

## Autonomic Systems

Despite the depths of knowledge in both vestibular and autonomic systems, still relatively little is known about vestibular-autonomic/cardiovascular reflexes. Cohen believed that there were a wide variety of opportunities to fill this gap. By applying OVAR to human subjects, Cohen et al. first established that signals that produce constriction of blood vessels in the legs can come from the vestibular system, namely the otolith organs, at a latency much shorter than baroreflex (81), a significant finding in the context where baroreflex is considered rapid as compared to chemoreceptor- or endocrine-mediated blood pressure regulation. They then established that trans-mastoidal electrical stimulation, presumably by activating the otoliths, can effectively activate sympathetic pathways with a similar short latency (82). Still, how blood pressure and heart rate might be affected by such galvanic vestibular stimulation was not known. When Cohen et al. applied galvanic vestibular stimulation to anesthetized rats, instead of an anticipated increase, a sudden decrease in blood pressure and heart rate, i.e., a vasovagal response, was elicited (83). As the diagnosis of vasovagal syncope is supported by a tilt-table test, which activates vestibular and body tilt senses, they hypothesized that the otoliths could provide significant input to the generation of syncope. Clearly, insights on otolith-mediated regulation of blood pressure were available to Cohen from space research as well. Cohen et al. had earlier suggested that otolith stimulation with centrifugation could be a countermeasure for post-flight orthostatic intolerance commonly seen among astronauts returning from space (80). Cohen forged ahead with studies of mechanisms of vasovagal response, which also demonstrated the usefulness of the small-animal model that he and his colleagues developed in the rat (84–88).

## Vestibular-Cerebellar Interaction

Early on, on the heels of his work with Suzuki on semicircular canal stimulation, Cohen, in the tradition of Bender, sought to determine patterns of eye movement represented in the cerebellum by correlating ocular responses to systematic stimulation of wide regions of the cerebellum (89). This work showed that the patterns of cerebellar-evoked eye movements resembled those produced by semicircular canal stimulation, that there were topographic separations for different eye movement planes, and that eye movements could be combined when two points were stimulated simultaneously. The work also suggested the possibility that, had the head not been fixed, postural movements might have been produced in place of eye movements at many points of stimulation, as well as paved the way for the idea that the brain may facilitate sensorimotor transformations for compensatory ocular and postural reflexes by using a common coordinate frame consistent with the arrangement of the semicircular canals (90), a point revisited by many authors (1, 91–96).

The cerebellar flocculus and nodulus are collectively known as the vestibulo-cerebellum for their close interactions with the vestibular system. Between the PPRF and velocity storage works, Cohen, with Takemori, showed that the flocculus aided rapid visual adjustment of the VOR (97), making a significant contribution to the field energized by Ito's hypothesis that placed the flocculus at the center of cerebellar motor control in the context of the VOR performance optimization (98).

With the formulation of velocity storage, the cerebellar control of the vestibular system fast became an essential consideration. To this effect, Cohen, with Waespe and Raphan, showed that the flocculus and the nodulus (together with the adjacent ventral uvula) have distinctive roles in vestibular control by demonstrating independent and model-predictable effects of floccular and nodular lesions on visual-vestibular interactions during the VOR, OKN/OKAN, and OVAR-generated nystagmus (42, 47, 99, 100)—in brief, the flocculus mediates the visual pathway with which the gain of the compensatory VOR is controlled, while the nodulus controls velocity storage. Cohen et al. further studied the nodulus to show that velocity storage dumping can be mimicked by electrical stimulation in the nodulus (101) and that the spatial orientation properties of velocity storage may be supported in relation to the parasagittal organization of the nodulus (102).

The thusly obtained knowledge had great implications in the approaches and clinical applications developed by Cohen and Mingjia Dai to motion sickness and mal de débarquement syndrome (MdDS). They postulated motion sickness as originating from a sensory conflict detected in the velocity storage mechanism, it being a focal point of multimodal sensory interaction and spatial orientation through its interactions with the nodulus and uvula (103–107). They then determined that, while velocity storage may be useful in other contexts, susceptibility to motion sickness could be reduced by *weakening* the velocity storage capacity in a targeted manner—reversibly by a pharmacological means with baclofen (108) or for a long-term effect by inducing a change in the VOR (109).

MdDS is still an under-recognized chronic balance disorder, characterized by persistent perception of oscillating self-motion, typically after coming off a cruise (110, 111). The condition, with additional likely secondary cognitive and affective symptoms, is debilitating. There is, as yet, not a fully effective treatment, but the approach developed recently at Mount Sinai has resulted in long-term improvement of symptoms in the majority of the treated patients (112, 113). The treatment was developed on the postulates that MdDS was a consequence of maladapted orientation properties of velocity storage (114) and that proper orientation properties could be restored through VOR re-adaptation. The success of the treatment was in many ways the culmination of Cohen's career. Interestingly, despite substantial evidence from Cohen's work that a change in the nodulus may have a significant behavioral consequence, the challenge remains to establish an experimental paradigm to explore plasticity in the nodulus amenable to cellular-level explanation.

## Conclusion

Cohen's scientific style was marked by enthusiasm. Besides engaging in diverse works related to and extending from the endeavors outlined above (115–118), he was ready to jump in whenever a scientific opportunity presented (119–123). At a tribute symposium held at Mount Sinai in April 2018 following Cohen's retirement from his long-held fulltime position as Morris B. Bender Professor of Neurology, Albert Fuchs of Washington University succinctly stated, “He was bitten by a science bug as a young man and never recovered from it.” He enjoyed good scientific battles like a proud prizefighter and inspired many with his spirit. Even after retirement, he continued to be deeply immersed in understanding neural mechanisms of vestibulosympathetic reflex, motion sickness, and MdDS. His scientific contributions will continue to drive research directions for many years to come.

## Author Contributions

The author confirms being the sole contributor of this work and has approved it for publication.

## Conflict of Interest

The author declares that the research was conducted in the absence of any commercial or financial relationships that could be construed as a potential conflict of interest.

## References

[1] Cohen B Raphan T The physiology of the vestibuloocular reflex (VOR). In: Highstein SM Fay RR Popper AM, editors. The Vestibular System. Springer Handbook of Auditory Research, vol 19. New York: Springer (2004). p. 235–85. 10.1007/0-387-21567-0_6

[2] Raphan T. Vestibular, locomotor, and vestibulo-autonomic research: 50 years of collaboration with Bernard Cohen. J Neurophysiol. (2020) 123:329–45. 10.1152/jn.00485.2019 31747361 PMC6985855

[3] Cohen B Lewis R Lopez-Escamez JA (eds.) Vestibular Contributions to Health and Disease. Lausanne: Frontiers Media (2018). 10.3389/978-2-88945-520-1 PMC5867307 29615952

[4] Cohen B Housepian EM Purpura DP. Intrathalamic regulation of activity in a cerebellocortical projection pathway. Exp Neurol. (1962) 6:492–506. 10.1016/0014-4886(62)90074-2 14022006

[5] Purpura DP Cohen B Marini G. Generalized neocortical responses and corticospinal neuron activity. Science. (1961) 134:729–30. 10.1126/science.134.3481.729 13738516

[6] Purpura DP Cohen B. Intracellular recording from thalamic neurons during recruiting responses. J Neurophysiol. (1962) 25:621–35. 10.1152/jn.1962.25.5.621 14489253

[7] Suzuki JI Cohen B Bender MB. Compensatory eye movements induced by vertical semicircular canal stimulation. Exp Neurol. (1964) 9:137–60. 10.1016/0014-4886(64)90013-5 14126123

[8] Cohen B Suzuki J Bender MB. Eye movements from semicircular canal nerve stimulation in the cat. Ann Otol Rhin Laryngol. (1964) 73:153–69. 10.1177/000348946407300116 14128701

[9] Suzuki JI Cohen B. Head, eye, body and limb movements from semicircular canal nerves. Exp Neurol. (1964) 10:393–405. 10.1016/0014-4886(64)90031-7 14228399

[10] Suzuki JI What is left behind in the valley? In: Kaga K, editor. The International Conference on the Visual and/versus Vestibular System; The Satellite Symposium to the IVth International Symposium on the Head/Neck System. Tokyo (1999). p. 33.

[11] Lorente de No R The regulation of eye positions and movements induced by the labyrinth: Chapter, V Rotation reflexes on the eye muscles. Laryngoscope. (1932) 42:298–312. 10.1288/00005537-193204000-00005

[12] Szentágothai J. The elementary vestibulo-ocular reflex arc. J Neurophysiol. (1950) 13:395–407. 10.1152/jn.1950.13.6.395 14784863

[13] Simpson JI Graf W. Eye-muscle geometry and compensatory eye movements in lateral-eyed and frontal-eyed animals. Ann N Y Acad Sci. (1981) 374:20–30. 10.1111/j.1749-6632.1981.tb30856.x 6978631

[14] Robinson DA. The use of matrices in analyzing the three-dimensional behavior of the vestibulo-ocular reflex. Biol Cybern. (1982) 46:53–66. 10.1007/bf00335351 6985203

[15] Highstein SM Holstein GR. The anatomy of the vestibular nuclei. Prog Brain Res. (2006) 151:157–203. 10.1016/S0079-6123(05)51006-9 16221589

[16] Della Santina CC Migliaccio AA Patel AH. A multichannel semicircular canal neural prosthesis using electrical stimulation to restore 3-d vestibular sensation. IEEE Trans Biomed Eng. (2007) 54:1016–30. 10.1109/TBME.2007.894629 17554821 PMC2767274

[17] Dlugaiczyk J Gensberger KD Straka H. Galvanic vestibular stimulation: from basic concepts to clinical applications. J Neurophysiol. (2019) 121:2237–55. 10.1152/jn.00035.2019 30995162

[18] Bender MB Shanzer S Oculomotor pathways defined by electric stimulation lesions in the brainstem of monkey. In Bender MB, editor. The Oculomotor System. New York: Harper and Row (1964). p. 81–140.

[19] Cohen B Komatsuzaki A Bender MB. Electrooculographic syndrome in monkeys after pontine reticular formation lesions. Arch Neurol. (1968) 18:78–92. 10.1001/archneur.1968.00470310092008 4964657

[20] Cohen B Feldman M. Relationship of electrical activity in pontine reticular formation and lateral geniculate body to rapid eye movements. J Neurophysiol. (1968) 31:806–17. 10.1152/jn.1968.31.6.806 4303956

[21] Cohen B Vestibulo-ocular relations. In: Bach-y-Rita P Collins CC, editoes. The Control of Eye Movements. New York/London: Academic Press (1971). p. 105–48.

[22] Cohen B Komatsuzaki A. Eye movements induced by stimulation of the pontine reticular formation: evidence for integration in oculomotor pathways. Exp Neurol. (1972) 36:101–17. 10.1016/0014-4886(72)90139-2 4558412

[23] Cohen B Henn V. Unit activity in the pontine reticular formation associated with eye movements. Brain Res. (1972) 46:403–10. 10.1016/0006-8993(72)90030-3 4628952

[24] Henn V Cohen B. Coding of information about rapid eye movements in the pontine reticular formation of alert monkeys. Brain Res. (1976) 108:307–25. 10.1016/0006-8993(76)90188-8 819098

[25] Robinson DA Integrating with neurons. Annu Rev Neurosci. (1989) 12:33–45. 10.1146/annurev.ne.12.030189.000341 2648952

[26] Cohen B Matsuo V Raphan T. Quantitative analysis of the velocity characteristics of optokinetic nystagmus and optokinetic after-nystagmus. J Physiol. (1977) 270:321–44. 10.1113/jphysiol.1977.sp011955 409838 PMC1353516

[27] ter Braak JWG Untersuchungen über optokinetischen Nystagmus. Arch Neerl Physiol. 21:309-376 (translated to English. Investigation on optokinetic nystagmus. In: Collewijn, H. The Oculomotor System of the Rabbit and Its Plasticity. Studies in Brain Function, Vol 5. Berlin; Heidelberg; New York, NY: Springer-Verlag (1936) p. 179-237).

[28] Collewijn H. An analog model of the rabbit's optokinetic system. Brain Res. (1972) 36:71–88. 10.1016/0006-8993(72)90767-6 5008386

[29] Raphan T Cohen B Matsuo V A velocity-storage mechanism responsible for optokinetic nystagmus (OKN), optokinetic after-nystagmus (OKAN) and vestibular nystagmus. In: Baker R. Berthoz A, editors. Control of Gaze by Brain Stem Neurons. Amsterdam/New York: Elsevier/North Holland Biomedical Press (1977). p. 37–47.

[30] Raphan T Matsuo V Cohen B. Velocity storage in the vestibulo-ocular reflex arc (VOR). Exp Brain Res. (1979) 35:229–48. 10.1007/BF00236613 108122

[31] Waespe W Henn V. Neuronal activity in the vestibular nuclei of the alert monkey during vestibular and optokinetic stimulation. Exp Brain Res. (1977) 27:523–38. 10.1007/BF00239041 404173

[32] Waespe W Henn V. Vestibular nuclei activity during optokinetic after-nystagmus (OKAN) in the alert monkey. Exp Brain Res. (1977) 30:323–30. 10.1007/BF00237259 413726

[33] Cohen B Uemura T Takemori S. Effects of labyrinthectomy on optokinetic nystagmus (OKN) and optokinetic after-nystagmus (OKAN). Equilib Res. (1973) 3:88–93. 4220148

[34] Collewijn H. Impairment of optokinetic (after-)nystagmus by labyrinthectomy in the rabbit. Exp Neurol. (1976) 52:146–56. 10.1016/0014-4886(76)90207-7 954907

[35] Robinson D Vestibular and optokinetic symbiosis: an example of explaining by modeling. In: Baker R Berthoz A, editors. Control of Gaze by Brain Stem Neurons. Amsterdam/New York: Elsevier/North Holland Biomedical Press (1977). p. 49–58.

[36] Dichgans J Brandt T Visual-vestibular interaction: effects on self-motion perception postural control. In: Held R Leibowitz HW Teuber HL, editors. Perception. Handbook of Sensory Physiology Vol. 8. Berlin; Heidelberg: Springer-Verlag (1978). p. 755–804. 10.1007/978-3-642-46354-9

[37] Bertolini G Ramat S Laurens J Bockisch CJ Marti S Straumann D. Velocity storage contribution to vestibular self-motion perception in healthy human subjects. J Neurophysiol. (2011) 105:209–23. 10.1152/jn.00154.2010 21068266

[38] Bertolini G Ramat S Bockisch CJ Marti S Straumann D Palla A. Is vestibular self-motion perception controlled by the velocity storage? Insights from patients with chronic degeneration of the vestibulo-cerebellum. PLoS ONE. (2012) 7:e36763. 10.1371/journal.pone.0036763 22719833 PMC3376140

[39] Reisine H Raphan T. Neural basis for eye velocity generation in the vestibular nuclei of alert monkeys during off-vertical axis rotation. Exp Brain Res. (1992) 92:209–26. 10.1007/BF00227966 1493862

[40] Yakushin SB Raphan T Cohen B. Coding of velocity storage in the vestibular nuclei. Front Neurol. (2017) 8:386. 10.3389/fneur.2017.00386 28861030 PMC5561016

[41] Raphan T Cohen B Henn V. Effects of gravity on rotatory nystagmus in monkeys. Ann N Y Acad Sci. (1981) 374:44–55. 10.1111/j.1749-6632.1981.tb30859.x 6978641

[42] Waespe W Cohen B Raphan T. Dynamic modification of the vestibulo-ocular reflex by the nodulus and uvula. Science. (1985) 228:199–202. 10.1126/science.3871968 3871968

[43] Cohen B Suzuki JI Raphan T. Role of the otolith organs in generation of horizontal nystagmus: effects of selective labyrinthine lesions. Brain Res. (1983) 276:159–64. 10.1016/0006-8993(83)90558-9 6626994

[44] Katz E de Jong JM Buettner-Ennever J Cohen B. Effects of midline medullary lesions on velocity storage and the vestibulo-ocular reflex. Exp Brain Res. (1991) 87:505–20. 10.1007/BF00227076 1783021

[45] Cohen B Helwig D Raphan T. Baclofen and velocity storage: a model of the effects of the drug on the vestibulo-ocular reflex in the rhesus monkey. J Physiol. (1987) 393:703–25. 10.1113/jphysiol.1987.sp016849 3446808 PMC1192419

[46] Jäger J Henn V. Habituation of the vestibulo-ocular reflex (VOR) in the monkey during sinusoidal rotation in the dark. Exp Brain Res. (1981) 41:108–14. 10.1007/BF00236599 6970677

[47] Cohen H Cohen B Raphan T Waespe W. Habituation and adaptation of the vestibuloocular reflex: a model of differential control by the vestibulocerebellum. Exp Brain Res. (1992) 90:526–38. 10.1007/BF00230935 1426111

[48] Raphan T Cohen B Suzuki J Henn V. Nystagmus generated by sinusoidal pitch while rotating. Brain Res. (1983) 276:165–72. 10.1016/0006-8993(83)90559-0 6626995

[49] Raphan T Dai M Maruta J Waespe W Henn V Suzuki JI. Canal and otolith afferent activity underlying eye velocity responses to pitching while rotating. Ann N Y Acad Sci. (1999) 871:181–94. 10.1111/j.1749-6632.1999.tb09184.x 10372071

[50] Hess BJ Angelaki DE. Angular velocity detection by head movements orthogonal to the plane of rotation. Exp Brain Res. (1993) 95:77–83. 10.1007/BF00229656 8405254

[51] Guedry FEJr. Orientation of the rotation-axis relative to gravity: its influence on nystagmus and the sensation of rotation. Acta Otolaryngol. (1965) 60:30–48. 10.3109/00016486509126986 14337956

[52] Benson AJ Bodin MA Interaction of linear and angular accelerations on vestibular receptors in man. Aerosp Med. (1966) 37:144–54. 5295433

[53] Correia MJ Money KE. The effect of blockage of all six semicircular canal ducts on nystagmus produced by dynamic linear acceleration in the cat. Acta Otolaryngol. (1970) 69:7–16. 10.3109/00016487009123331 5310027

[54] Guedry FE Jr Benson AJ. Coriolis cross-coupling effects: disorienting and nauseogenic or not? Aviat Space Environ Med. (1978) 49:29–35. 304719

[55] Maruta J Simpson JI Raphan T Cohen B. Orienting eye movements and nystagmus produced by translation while rotating (TWR). Exp Brain Res. (2005) 163:273–83. 10.1007/s00221-004-2178-5 15702320

[56] Raphan T Cohen B Multidimensional organization of the vestibulo-ocular reflex (VOR). In Keller EL Zee DS editors. Adaptive Processes in Visual and Oculomotor Systems. Oxford: Pergamon Press (1986). p. 285-92.

[57] Raphan T Cohen B. Organizational principles of velocity storage in three dimensions: the effect of gravity on cross-coupling of optokinetic after-nystagmus. Ann N Y Acad Sci. (1988) 545:74–92. 10.1111/j.1749-6632.1988.tb19556.x 3239884

[58] Schiff D Cohen B Raphan T Nystagmus induced by stimulation of the nucleus of the optic tract in the monkey. Exp Brain Res. (1988) 70:1–14. 10.1007/BF00271841 3261253

[59] Wearne S Raphan T Cohen B. Contribution of vestibular commissural pathways to spatial orientation of the angular vestibuloocular reflex. J Neurophysiol. (1997) 78:1193–7. 10.1152/jn.1997.78.2.1193 9307151

[60] Raphan T Dai M Cohen B. Spatial orientation of the vestibular system. Ann N Y Acad Sci. (1992) 656:140–57. 10.1111/j.1749-6632.1992.tb25205.x 1599139

[61] Dai MJ Raphan T Cohen B. Spatial orientation of the vestibular system: dependence of optokinetic after-nystagmus on gravity. J Neurophysiol. (1991) 66:1422–39. 10.1152/jn.1991.66.4.1422 1761991

[62] Raphan T Sturm D. Modeling the spatiotemporal organization of velocity storage in the vestibuloocular reflex by optokinetic studies. J Neurophysiol. (1991) 66:1410–21. 10.1152/jn.1991.66.4.1410 1761990

[63] Cohen B Wearne S Dai M Raphan T. Spatial orientation of the angular vestibulo-ocular reflex. J Vestib Res. (1999) 9:163–72. 10436469

[64] Cohen B Dai M Raphan T. The critical role of velocity storage in production of motion sickness. Ann N Y Acad Sci. (2003) 1004:359–76. 10.1196/annals.1303.034 14662476

[65] Raphan T Cohen B How does the vestibulo-ocular reflex work? In: Baloh R Halmagyi GM, editors. Disorders of the Vestibular System. New York: Oxford University Press (1996). p. 20–47.

[66] Schöne H Spatial Orientation. Princeton, NJ: Princeton University Press (1984).

[67] Brandt T Büchele W Arnold F. Arthrokinetic nystagmus and ego-motion sensation. Exp Brain Res. (1977) 30:331–8. 10.1007/BF00237260 598431

[68] Bles W de Jong JM de Wit G. Somatosensory compensation for loss of labyrinthine function. Acta Otolaryngol. (1984) 97:213–21. 10.3109/00016488409130982 6609519

[69] Solomon D Cohen B Stabilization of gaze during circular locomotion in light. I Compensatory head and eye nystagmus in the running monkey. J Neurophysiol. (1992) 67:1146–57. 10.1152/jn.1992.67.5.1146 1597704

[70] Hirasaki E Moore ST Raphan T Cohen B. Effects of walking velocity on vertical head and body movements during locomotion. Exp Brain Res. (1999) 127:117–30. 10.1007/s002210050781 10442403

[71] Solomon D Cohen B. Stabilization of gaze during circular locomotion in darkness. II Contribution of velocity storage to compensatory eye and head nystagmus in the running monkey. J Neurophysiol. (1992) 67:1158–70. 10.1152/jn.1992.67.5.1158 1597705

[72] Raphan T Cohen B. The vestibulo-ocular reflex in three dimensions. Exp Brain Res. (2002) 145:1–27. 10.1007/s00221-002-1067-z 12070741

[73] Cohen B Maruta J Raphan T Orientation of the eyes to gravitoinertial acceleration. Ann N Y Acad Sci. (2001) 942:241–58. 10.1111/j.1749-6632.2001.tb03750.x 11710466

[74] Imai T Moore ST Raphan T Cohen B. Interaction of the body, head, and eyes during walking and turning. Exp Brain Res. (2001) 136:1–18. 10.1007/s002210000533 11204402

[75] Dai M Raphan T Kozlovskaya I Cohen B. Vestibular adaptation to space in monkeys. Otolaryngol Head Neck Surg. (1998) 119:65–77. 10.1016/S0194-5998(98)70175-5 9674517

[76] Highstein SM Cohen B. Neurolab mission. Curr Opin Neurobiol. (1999) 1999:495–9. 10.1016/s0959-4388(99)80074-9 10448169

[77] Clément G Moore ST Raphan T Cohen B. Perception of tilt (somatogravic illusion) in response to sustained linear acceleration during space flight. Exp Brain Res. (2001) 138:410–8. 10.1007/s002210100706 11465738

[78] Moore ST Clément G Raphan T Cohen B. Ocular counterrolling induced by centrifugation during orbital space flight. Exp Brain Res. (2001) 137:323–35. 10.1007/s002210000669 11355379

[79] Moore ST Cohen B Raphan T Berthoz A Clément G. Spatial orientation of optokinetic nystagmus and ocular pursuit during orbital space flight. Exp Brain Res. (2005) 160:38–59. 10.1007/s00221-004-1984-0 15289967

[80] Moore ST Diedrich A Biaggioni I Kaufmann H Raphan T Cohen B. Artificial gravity: a possible countermeasure for post-flight orthostatic intolerance. Acta Astronaut. (2005) 56:867–76. 10.1016/j.actaastro.2005.01.012 15835033

[81] Kaufmann H Biaggioni I Voustianiouk A Diedrich A Costa F Clarke R. Vestibular control of sympathetic activity. An otolith-sympathetic reflex in humans. Exp Brain Res. (2002) 143:463–9. 10.1007/s00221-002-1002-3 11914792

[82] Voustianiouk A Kaufmann H Diedrich A Raphan T Biaggioni I Macdougall H. Electrical activation of the human vestibulo-sympathetic reflex. Exp Brain Res. (2006) 171:251–61. 10.1007/s00221-005-0266-9 16308690

[83] Cohen B Martinelli GP Ogorodnikov D Xiang Y Raphan T Holstein GR. Sinusoidal galvanic vestibular stimulation (sGVS) induces a vasovagal response in the rat. Exp Brain Res. (2011) 210:45–55. 10.1007/s00221-011-2604-4 21374078 PMC3134240

[84] Holstein GR Friedrich VL Jr Martinelli GP Ogorodnikov D Yakushin SB Cohen B. Fos expression in neurons of the rat vestibulo-autonomic pathway activated by sinusoidal galvanic vestibular stimulation. Front Neurol. (2012) 3:4. 10.3389/fneur.2012.00004 22403566 PMC3289126

[85] Cohen B Martinelli GP Raphan T Schaffner A Xiang Y Holstein GR. The vasovagal response of the rat: its relation to the vestibulosympathetic reflex and to Mayer waves. FASEB J. (2013) 27:2564–72. 10.1096/fj.12-226381 23504712 PMC3688754

[86] Cohen B Martinelli GP Xiang Y Raphan T Yakushin SB. Vestibular activation habituates the vasovagal response in the rat. Front Neurol. (2017) 8:83. 10.3389/fneur.2017.00083 28360882 PMC5350135

[87] Yakushin SB Martinelli GP Raphan T Xiang Y Holstein GR Cohen B. Vasovagal oscillations and vasovagal responses produced by the vestibulo-sympathetic reflex in the rat. Front Neurol. (2014) 5:37. 10.3389/fneur.2014.00037 24772102 PMC3983498

[88] Raphan T Cohen B Xiang Y Yakushin SB. A Model of blood pressure, heart rate, and vaso-vagal responses produced by vestibulo-sympathetic activation. Front Neurosci. (2016) 10:96. 10.3389/fnins.2016.00096 27065779 PMC4814511

[89] Cohen B Goto K Shanzer S Weiss AH. Eye movements induced by electric stimulation of the cerebellum in the alert cat. Exp Neurol. (1965) 13:145–62. 10.1016/0014-4886(65)90105-6 5320100

[90] Graf W Simpson JI Leonard CS Spatial organization of visual messages of the rabbit's cerebellar flocculus. II Complex and simple spike responses of Purkinje cells. J Neurophysiol. (1988) 60:2091–121. 10.1152/jn.1988.60.6.2091 3236063

[91] Graf W Wilson VJ. Afferents and efferents of the vestibular nuclei: the necessity of context-specific interpretation. Prog Brain Res. (1989) 80:149–57. 10.1016/s0079-6123(08)62208-6 2699362

[92] Masino T Knudsen EI. Horizontal and vertical components of head movement are controlled by distinct neural circuits in the barn owl. Nature. (1990) 345:434–7. 10.1038/345434a0 2342573

[93] Hess BJ Dieringer N. Spatial organization of linear vestibuloocular reflexes of the rat: responses during horizontal and vertical linear acceleration. J Neurophysiol. (1991) 66:1805–18. 10.1152/jn.1991.66.6.1805 1812218

[94] Wylie DR Bischof WF Frost BJ. Common reference frame for neural coding of translational and rotational optic flow. Nature. (1998) 392:278–82. 10.1038/32648 9521321

[95] Angelaki DE Yakusheva TA Green AM Dickman JD Blazquez PM. Computation of egomotion in the macaque cerebellar vermis. Cerebellum. (2010) 9:174–82. 10.1007/s12311-009-0147-z 20012388 PMC3640361

[96] Branoner F Straka H. Semicircular canal influences on the developmental tuning of the translational vestibulo-ocular reflex. Front Neurol. (2018) 9:404. 10.3389/fneur.2018.00404 29922219 PMC5996107

[97] Takemori S Cohen B. Loss of visual suppression of vestibular nystagmus after flocculus lesions. Brain Res. (1974) 72:213–24. 10.1016/0006-8993(74)90860-9 4209081

[98] Ito M. Neural design of the cerebellar motor control system. Brain Res. (1972) 40:81–4. 10.1016/0006-8993(72)90110-2 4338265

[99] Waespe W Cohen B Raphan T. Role of the flocculus and paraflocculus in optokinetic nystagmus and visual-vestibular interactions: effects of lesions. Exp Brain Res. (1983) 50:9–33. 10.1007/BF00238229 6357831

[100] Waespe W Cohen B. Flocculectomy and unit activity in the vestibular nuclei during visual-vestibular interactions. Exp Brain Res. (1983) 51:23–35. 10.1007/BF00236799 6884464

[101] Solomon D Cohen B. Stimulation of the nodulus and uvula discharges velocity storage in the vestibulo-ocular reflex. Exp Brain Res. (1994) 102:57–68. 10.1007/BF00232438 7895799

[102] Wearne S Raphan T Cohen B. Control of spatial orientation of the angular vestibuloocular reflex by the nodulus and uvula. J Neurophysiol. (1998) 79:2690–715. 10.1152/jn.1998.79.5.2690 9582239

[103] Dai M Kunin M Raphan T Cohen B. The relation of motion sickness to the spatial-temporal properties of velocity storage. Exp Brain Res. (2003) 151:173–89. 10.1007/s00221-003-1479-4 12783152

[104] Dai M Raphan T Cohen B. Labyrinthine lesions and motion sickness susceptibility. Exp Brain Res. (2007) 178:477–87. 10.1007/s00221-006-0759-1 17256169 PMC3181155

[105] Dai M Sofroniou S Kunin M Raphan T Cohen B. Motion sickness induced by off-vertical axis rotation (OVAR). Exp Brain Res. (2010) 204:207–22. 10.1007/s00221-010-2305-4 20535456 PMC3181161

[106] Cohen B Dai M Ogorodnikov D Laurens J Raphan T Müller P. Motion sickness on tilting trains. FASEB J. (2011) 25:3765–74. 10.1096/fj.11-184887 21788449 PMC3205836

[107] Cohen B Dai M Yakushin SB Cho C. The neural basis of motion sickness. J Neurophysiol. (2019) 121:973–82. 10.1152/jn.00674.2018 30699041

[108] Cohen B Dai M Yakushin SB Raphan T Baclofen, motion sickness susceptibility and the neural basis for velocity storage. Prog Brain Res. (2008) 171:543–53. 10.1016/S0079-6123(08)00677-8 18718351 PMC12964195

[109] Dai M Raphan T Cohen B. Prolonged reduction of motion sickness sensitivity by visual-vestibular interaction. Exp Brain Res. (2011) 210:503–13. 10.1007/s00221-011-2548-8 21287155 PMC3182575

[110] Brown JJ Baloh RW. Persistent mal de debarquement syndrome: a motion-induced subjective disorder of balance. Am J Otolaryngol. (1987) 8:219–22. 10.1016/s0196-0709(87)80007-8 3631419

[111] Cha YH. Mal de debarquement. Semin Neurol. (2009) 29:520–7. 10.1055/s-0029-1241038 19834863 PMC2846419

[112] Dai M Cohen B Smouha E Cho C. Readaptation of the vestibulo-ocular reflex relieves the mal de debarquement syndrome. Front Neurol. (2014) 5:124. 10.3389/fneur.2014.00124 25076935 PMC4097942

[113] Dai M Cohen B Cho C Shin S Yakushin SB. Treatment of the mal de debarquement syndrome: a 1-year follow-up. Front Neurol. (2017) 8:175. 10.3389/fneur.2017.00175 28529496 PMC5418223

[114] Dai M Raphan T Cohen B. Adaptation of the angular vestibulo-ocular reflex to head movements in rotating frames of reference. Exp Brain Res. (2009) 195:553–67. 10.1007/s00221-009-1825-2 19458941

[115] Cohen B Büttner-Ennever JA. Projections from the superior colliculus to a region of the central mesencephalic reticular formation (cMRF) associated with horizontal saccadic eye movements. Exp Brain Res. (1984) 57:167–76. 10.1007/BF00231143 6519224

[116] Waitzman DM Silakov VL Cohen B. Central mesencephalic reticular formation (cMRF) neurons discharging before and during eye movements. J Neurophysiol. (1996) 75:1546–72. 10.1152/jn.1996.75.4.1546 8727396

[117] Holstein GR Martinelli GP Cohen B. L-baclofen-sensitive GABAB binding sites in the medial vestibular nucleus localized by immunocytochemistry. Brain Res. (1992) 581:175–80. 10.1016/0006-8993(92)90361-c 1323367

[118] Holstein GR Martinelli GP Cohen B The ultrastructure of GABA-immunoreactive vestibular commissural neurons related to velocity storage in the monkey. Neuroscience. (1999) 93:171–81. 10.1016/s0306-4522(99)00141-4 10430481

[119] de Jong PT de Jong JM Cohen B Jongkees LB. Ataxia and nystagmus induced by injection of local anesthetics in the neck. Ann Neurol. (1977) 1:240–6. 10.1002/ana.410010307 407834

[120] Bala SP Cohen B Morris AG Atkin A Gittelman R Kates W. Saccades of hyperactive and normal boys during ocular pursuit. Dev Med Child Neurol. (1981) 23:323–36. 7250541

[121] Cohen G Pasik P Cohen B Leist A Mytilineou C Yahr MD. Pargyline and deprenyl prevent the neurotoxicity of 1-methyl-4-phenyl-1,2,3,6-tetrahydropyridine (MPTP) in monkeys. Eur J Pharmacol. (1984) 106:209–10. 10.1016/0014-2999(84)90700-3 6442232

[122] Büttner-Ennever JA Horn AK Henn V Cohen B. Projections from the superior colliculus motor map to omnipause neurons in monkey. J Comp Neurol. (1999) 413:55–67. 10.1002/(sici)1096-9861(19991011)413:1<55::aid-cne3>3.0.co;2-k 10464369

[123] Rucker JC Buettner-Ennever JA Straumann D Cohen B. Case studies in neuroscience: instability of the visual near triad in traumatic brain injury-evidence for a putative convergence integrator. J Neurophysiol. (2019) 122:1254–63. 10.1152/jn.00861.2018 31339793

